# Enzymatic Hydrolysis and Simultaneous Saccharification and Fermentation of Soybean Processing Intermediates for the Production of Ethanol and Concentration of Protein and Lipids

**DOI:** 10.5402/2012/278092

**Published:** 2012-10-17

**Authors:** Craig C. Long, William Gibbons

**Affiliations:** Department of Biology and Microbiology, South Dakota State University, Brookings, SD 57007, USA

## Abstract

Carbohydrates in soybeans are generally undesirable due to their low digestibility and because they “dilute” more valuable components (proteins, lipids). To remove these carbohydrates and raise the titer of more valuable components, ethanol production was investigated. Commercial enzymes (Novozyme cellulase, **β**-glucosidase, and pectinase) were added to ground soybeans (SB), soybean meal (SBM), soybean hulls (SH), and soybean white flakes (WF) at a 10% solids loading rate to quantify hydrolyzed glucan. Saccharification resulted in glucan reductions of 28%, 45%, 76%, and 80% (SBM, SB, SH, WF, resp.). Simultaneous saccharification and fermentation (SSF) trials were conducted at 5%, 10%, 15%, and 20% solids loading with *Saccharomyces cerevisiae *NRRL Y-2034 and *Scheffersomyces stipitis *NRRL Y-7124, with protein, fiber, and lipids analyzed at SSF 10% solids and saccharification trials. *S. cerevisiae *and *S. stipitis *produced ~3–12.5 g/L ethanol and ~2.5–8.6 g/L ethanol, respectively, on SB, SBM, and WF over all solid loading rates. SH resulted in higher ethanol titers for both *S. cerevisiae *(~9–23 g/L) and *S. stipitis *(~9.5–14.5 g/L). Protein concentrations decreased by 2.5–10% for the SB, SBM, and WF, but increased by 53%–55% in SH. Oil concentrations increased by ~50% for SB; by ~500%–1300% for the others.

## 1. Introduction

Soybeans are one of the most valuable crops in the world due to their high oil and protein content, which provides for a wide variety of uses. Soybean oil is used as a food and feed ingredient as well as in cosmetics [1–4] and biodiesel production [5]. Soybean protein is highly digestible and has been used in livestock and aquaculture feeds, along with many human foods [6–10]. Soybean protein supplements are promoted in human diets due to their many health benefits [9, 11–15].

In contrast to the oil and proteins, carbohydrates found in soybeans, (~10% dry weight), are largely undesirable due to their low digestibility [16]. Stachyose and raffinose, two of the primary carbohydrates in soybeans, are indigestible by humans and other monogastrics but can be fermented by natural flora in the intestinal tract, causing discomforting gas buildup [17–19]. Stachyose and raffinose may also decrease the digestibility of foods which contain them [17]. The presence of these carbohydrates also effectively dilutes the concentration of the protein and oil in soybeans.

In many countries, soybeans are “crushed” and then extracted with hexane or other solvents to separate soybean oil from the solids (i.e., soybean meal, SBM). SBM has a protein content of about 45% and is widely used as a livestock feed. Soybeans can be further processed via ethanol extraction to remove carbohydrates, resulting in soy protein concentrate (SPC) that contains at least 65% protein [18]. This is an expensive process and the removed sugars have little use, but the SPC is much more digestible and commands a price 2–2.5 times that of SBM.

As an alternative to ethanol washing, we evaluated saccharification and bioconversion of soybean carbohydrates to ethanol. This would create an additional product to help offset processing costs, while making use of an underutilized material (soybean carbohydrates). Commercial hydrolytic enzymes and yeast were tested on soybeans and three fractions from the soybeanoil extraction facility. We hypothesized that much of the protein or lipids used by yeast as nutrients would be left in the final solids as yeast cell mass. Moreover, we expected an increase in protein content due to the conversion of carbohydrates into cell protein.

## 2. Materials and Methods

### 2.1. Substrates

**Figure 1:**
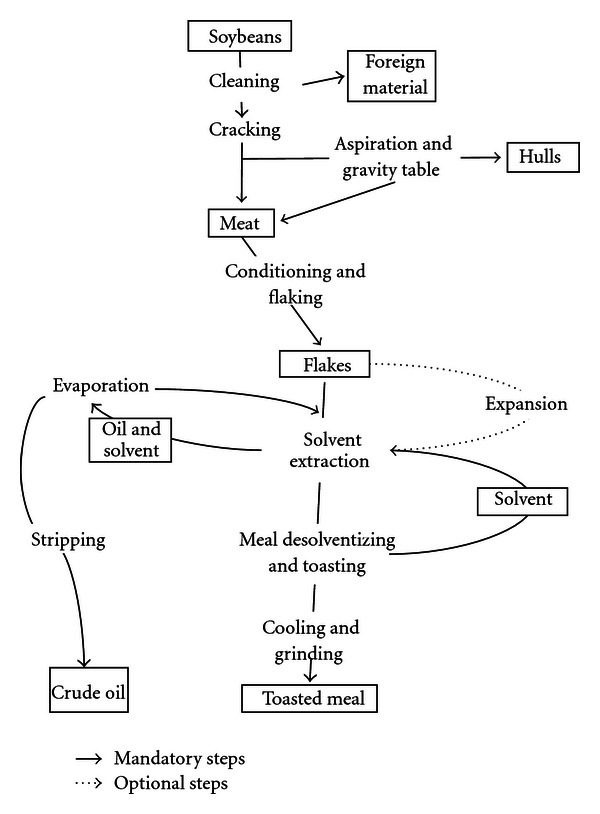
Organic solvent soybean processing [20].

Substrates tested included whole soybeans, soybean hulls, white flakes, and defatted soybean meal. These were obtained as a gift from South Dakota Soybean Processors, Volga, SD, USA.  Figure 1 shows a simplified process flow diagram of organic-solvent soybean processing to denote the source of the substrates. The substrates were ground using a Wiley Mill (2 mm) screen and stored at room temperature. These substrates were subjected to proximate analysis by Olson Agricultural Analytical Laboratories at South Dakota State University in Brookings, SD, USA and the results are shown in Table 1.

### 2.2. Enzymes

Enzymes were obtained as a gift from Novozymes (Franklinton, NC, USA). NS 50013 (Celluclast 1.5 L) is a cellulase cocktail with an activity of 70 FPU/g. NS 50010 (Novozyme 188) is a *β*-glucosidase with an activity of 250 CBU/g. NS 22016 is a pectinase cocktail with an activity of 3800 U/mL. Enzymes were stored at 4°C prior to use.

### 2.3. Yeast

*Saccharomyces cerevisiae *NRRL Y-2034 and *Scheffersomyces stipitis* NRRL Y-7124 were obtained from the USDA ARS Culture Collection (Peoria, IL, USA). For short-term maintenance, cultures were grown on Potato Dextrose Agar (PDA) plates and slants for 72 h at 35°C and then stored at 4°C, with subculturing of the organisms every 4 weeks. Lyophilization in a 20% sucrose solution was used for long-term storage. 

Inoculum for all trials was prepared by aseptically inoculating sterile 5% glucose, 0.5% yeast extract broth (100 mL in a 250 mL Erlenmeyer flasks) with a 1% (v/v) aliquot for *S. cerevisiae*, or 5% (v/v) for *S. stipitis*, from broth seed cultures stored at 4°C. Flasks for inoculum were incubated for 24 h at 35°C in a 250 rpm rotary shaker. Broth seed cultures were grown for 24 h at 250 rpm before refrigeration and used within 60 days to inoculate flasks for inoculum.

### 2.4. Buffers and Antibiotics

Saccharification and SSF trials were conducted in a sterile 0.1 M sodium citrate buffer with the pH adjusted to 4.8 using concentrated H_2_SO_4_. A stock solution of 10 mg/mL tetracycline HCl (70% ethanol and filter-sterilized) was prepared and stored at −20°C, from which 0.4 mL/100 mL of total trial volume was used to prevent bacterial contamination. A stock solution of 10 mg/mL cycloheximide (filter-sterilized) was prepared and stored at 4°C, from which 0.3 mL/100 mL of total trial volume was used for contamination control in saccharification trials only.

### 2.5. Saccharification of Soybean Fractions

Saccharification trials were conducted by mixing 15 g of ground substrate with 75 mL of sterile 0.1 M sodium citrate buffer, along with tetracycline and cycloheximide solutions in 250 mL Erlenmeyer flasks fitted with rubber stoppers. The pH of the solutions was adjusted to 5.0 using concentrated H_2_SO_4_ or 12 M NaOH. The stoppers were pierced with 21 gauge syringe needles and Whatman 0.2 *μ*m syringe filters. Enzyme dosages per gram of glucan included 23.2 FPU of NS 50013, 41 CBU of NS50010, and 500 U NS 22016.  Table 2 provides a summary of glucan levels found in the literature for each soybean fraction, and these were averaged to calculate enzyme dosage.  Table 3 shows the volume of enzymes used for each substrate. Sterile-deionized water was added to each flask to bring the total volume to 150 mL, resulting in a solid loading rate of 10%. Saccharification trials were run for 96 h in a 50°C reciprocating shaker set at 250 rpm. Flasks lacking enzymes were used as controls to determine the type and amount of carbohydrates that would be released by solubilization. 

### 2.6. Simultaneous Saccharification and Fermentation of Soybean Fractions

Ground substrates (15, 30, 45, or 60 g) were mixed with 150 mL of sterile 0.1 M sodium citrate buffer, along with an appropriate amount of tetracycline solution in 500 mL Erlenmeyer flasks fitted with rubber stoppers that were pierced with 21 gauge syringe needles and attached to Whatman 0.2 *μ*m syringe filters. The pH was adjusted to 5.0 using concentrated H_2_SO_4_ or 12 M NaOH. The substrates were not autoclaved in an effort to preserve protein and carbohydrate integrity by preventing the Maillard reaction [25] as well as retaining the most likely parameters for industrial applications. Enzyme dosages per gram of glucan included 23.2 FPU of NS 50013, 41 CBU of NS 50010, and 500 U of NS 22016.  Table 2 shows the amount of substrate, fiber, and enzymes used at the 5% loading rate for each substrate. Amounts of these components increased proportionally at the higher solid loading levels. Sterile-deionized water was added to bring the total volume to 297 mL, and then 3 mL of a 24 h culture of either *S. cerevisiae *or *S. stipitis *was added. Flasks were incubated for 96 h in a 35°C reciprocating shaker set at 250 rpm. Control flasks without enzymes were also included to assess ethanol production from the free carbohydrates released from the substrates. These controls were prepared in the same manner as described above, except that the volumes of enzymes were replaced with sterile-deionized water.

### 2.7. Analytical Methods

Samples (5–10 mL) were drawn aseptically from the flasks at 0, 2, 4, 6, 12, 24, 48, 72, and 96 h. Samples were boiled for 5 minutes to inactivate enzymes and then centrifuged at 2400 ×g for 10 min. After freezing for 24 h at −20°C, samples were thawed, centrifuged again at 13,000 rpm for 15 min, and the supernatant was filtered through 0.2 *μ*m syringe filter into HPLC autosampler vials.

Carbohydrates were analyzed using a Waters 1200 HPLC with a Waters Sugar-pak I column and refractive index detector. The mobile phase for the Sugar-pak I column was 0.0001 M calcium EDTA flowing at a rate of 0.5 mL/min, with the column at 65°C. Ethanol concentrations were determined using a Waters 717 HPLC with an Aminex HPX-87H column and Waters 2410 refractive index detector (RID). The mobile phase was 0.005 M H_2_SO_4_ flowing at a rate of 0.6 mL/min, with the column at 65°C. 

At the end of saccharification and SSF trials, in the 10% solid loading rate experiments, the slurries from all replicates of a trial were combined and evaporated to dryness in an 80°C oven for 96 h. A proximate analysis was performed on the solids by Olson Agricultural Analytical Laboratory Services (South Dakota State University, Brookings, SD, USA).

### 2.8. Data Analysis

Saccharification trials were done in replicates of six, while the SSF trials were done in triplicate. Parameters analyzed included maximum ethanol concentration, ethanol productivity, and residual carbohydrates. The percent difference of the fiber, protein, and lipid content when compared to the original substrate was calculated using the formulas listed below. Residual carbohydrates were corrected by subtracting additional carbohydrate results that resulted from denatured enzymes or buffer. Graphs and calculations were made in Microsoft Excel 2007.

- Ethanol Productivity (g/L/h) = (Net Maximum Ethanol Concentration)/Time.
- Component Percent Difference (%) = ((% of dried slurry after trial) − (% of original substrate))/% of original substrate.

## 3. Results and Discussion

### 3.1. Saccharification of Soybean Fractions

Table 4 shows the composition of the four soybean substrates following 96 h saccharification. Trials (six replicates) were conducted at the 10% solid loading rate, both with and without enzymes. Soluble carbohydrate levels in the saccharified broth were determined via HPLC for each individual replication. Following saccharification, solids from the six replications of each treatment were combined, dried, and analyzed for fiber, protein, and lipid levels. The percentage difference for the fiber, protein, and lipid concentrations, compared to the substrate before saccharification, was calculated.

As expected, the presence of enzymes resulted in higher soluble carbohydrate levels for each of the substrates, compared to control trials lacking enzymes. This difference was statistically significant for all substrates except white flakes and was also correlated with the reduction in fiber content. Whole beans contained the lowest concentration of fiber, due to the presence of both lipids and protein. Consequently, soluble carbohydrates were the lowest and only a moderate reduction in percent fiber was observed. Enzymatic saccharification efficiency in raw beans may have been reduced by the lack of any pretreatment effect that the typical soy processing operation provides. On the other hand, hulls contained the highest level of fiber and therefore responded most significantly to enzymatic hydrolysis, yielding the highest level of carbohydrates and greatest percent reduction in fiber.

In the soybean crushing process, after oil extraction the solids are referred to as white flake. This material is then heated to drive off any residual hexane and inactivate certain antinutritional factors. Low levels of hulls then may be added to white flake to reduce the protein content to ~45%. This material is then called soybean meal. Thus, white flake and soybean meal are relatively similar in composition and we anticipated similar results upon saccharification. As can be seen in Table 4, soybean meal resulted in higher soluble carbohydrates and a greater effect of enzyme addition. Perhaps this was due to the additional heat treatment and/or presence of some hulls.

Protein and lipid levels in the samples were not expected to change significantly, since only small amounts of enzymes, buffers, and other components were added and the total solids were recovered. The only significant change expected was the conversion of fiber to soluble sugars as described above. Changes in relative protein levels varied from −14% to +11% and showed no significant trends. Lipid levels in whole beans increased from 4.5% to 12.9%, again likely due to greater solubilization during the saccharification process. Lipid levels in the other materials were very low, and therefore slight variability in values resulted in large percent changes. 

### 3.2. Soybean Substrate SSF

Each substrate was subjected to SSF treatment using four different concentrations of substrate (5%, 10%, 15%, or 20% solid loading rate (SLR)) as well as either *S. cerevisiae* or *S. stipitis*. Enzyme dosages were normalized based on glucan levels and control trials lacking enzymes were also performed. Carbohydrate and ethanol titers were monitored throughout each 96 h SSF. After SSF, the replicates from the 10% solid loading rate treatments were combined and dehydrated for fiber, protein, and lipid analysis by Olson Agricultural Analytical Laboratory Services.

**Figure 2:**
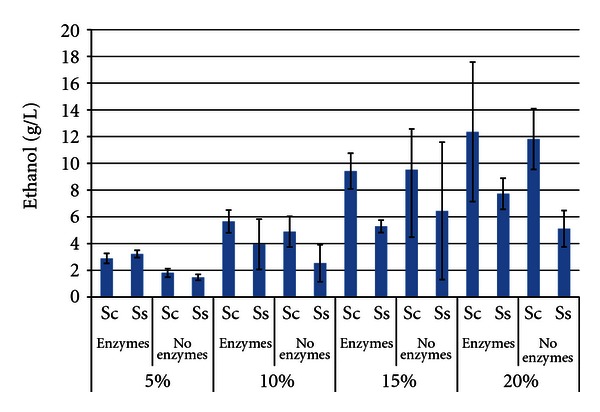
Maximum ethanol titer from soybeans after 96 h SSF or fermentation^1^. ^1^Error bars represent one standard deviation.

Figure 2 shows that the maximum ethanol titer of both yeasts increased as the SLR of soybeans was increased from 5% to 20%, as was expected. In most treatments, *S. cerevisiae* produced more ethanol than *S. stipitis *(maxima of 12.357 g/L ± 5.213 for *S. cerevisiae* with enzymes, 7.726 g/L ± 1.167 for *S. stipitis*). However, the difference was only significant in the 15% SLR trial with enzymes and the 20% SLR trial without enzymes. The presence of enzymes enhanced ethanol levels, but was only statistically significant in the 5% SLR trials. The high degree of variability in the 15% and 20% SLR trials was likely due to the high viscosity of these trials, which reduced mixing efficiency. 

**Figure 3:**
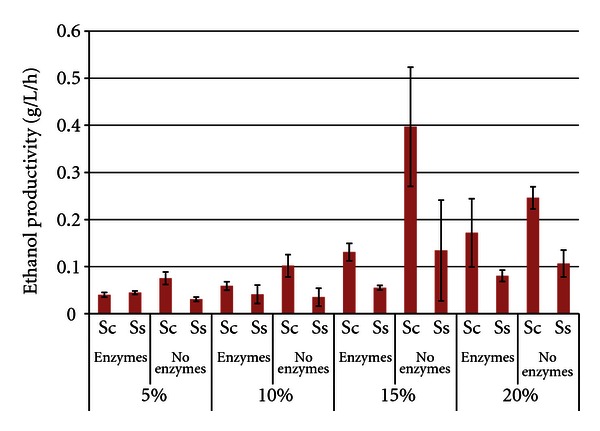
Ethanol productivity from soybeans after 96 h SSF or fermentation^1^. ^1^Error bars represent one standard deviation.

Figure 3 shows the corresponding ethanol productivities for the soybean SSF or fermentation trials, which were calculated at the time of maximum ethanol concentration. As expected, ethanol productivities also increased as SLRs increased from 5% to 20%. In most comparisons, *S. cerevisiae* had significantly higher productivities compared to *S. stipitis*. Ethanol productivities were actually higher in many of the enzyme-free trials (maxima of 0.397 g/L/h ± 0.127 for *S. cerevisiae* and 0.134 g/L/h ± 0.107 for *S. stipitis*, both 15% SLR without enzymes), suggesting that enzymatic hydrolysis of the nonpretreated soybean was the rate limiting factor. 

**Figure 4:**
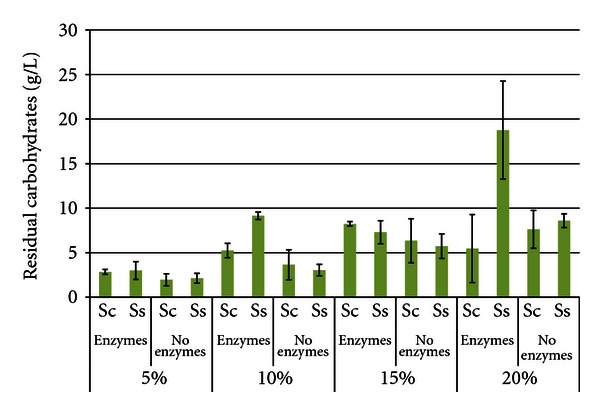
Residual carbohydrates from soybeans after 96 h SSF or fermentation^1^. ^1^Error bars represent one standard deviation.

Figure 4 shows the total residual carbohydrate levels after 96 h SSF or fermentation. The levels of residual carbohydrates increased as the SLR increased, reflecting an accumulation of stachyose, raffinose, or partial hydrolysis products of the oligosaccharides. This was due to the inability of either yeast to fully catabolize the oligosaccharides [26]. *S. cerevisiae* can hydrolyze the fructose residue from both stachyose and raffinose by use of invertase [27, 28], but cannot hydrolyze the other bonds. *S. stipitis *does not produce invertase and cannot catabolize either oligosaccharide. In most treatments, total residual carbohydrate levels were similar between yeasts; however, at the 10% and 20% SLR trials with enzymes, *S. stipitis* accumulated significantly higher carbohydrate levels than *S. cerevisiae*. Also expected were the higher carbohydrate levels in enzyme-hydrolyzed trials compared to enzyme-free trials. The highest carbohydrate concentration was 20% SLR with enzymes and *S. stipitis* (18.76 g/L ± 5.501), and the lowest was 5% SLR without enzymes with *S. cerevisiae *(1.96 g/L ± 0.661).

**Figure 5:**
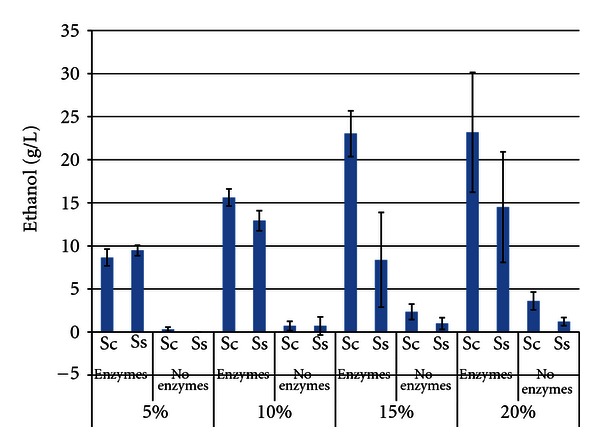
Maximum ethanol titer from hulls after 96 h SSF or fermentation^1^. ^1^Error bars represent one standard deviation.

**Figure 6:**
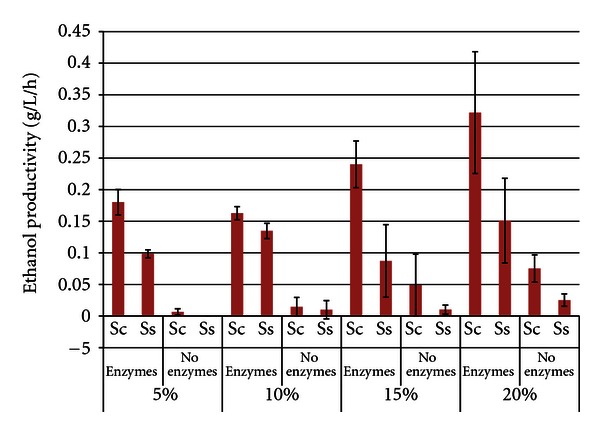
Ethanol productivity from hulls after 96 h SSF or fermentation^1^. ^1^Error bars represent one standard deviation.

**Figure 7:**
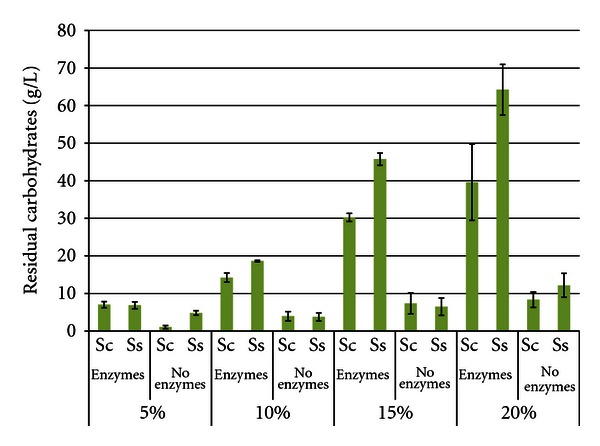
Residual carbohydrates from hulls after 96 h SSF or fermentation^1^. ^1^Error bars represent one standard deviation.

Figures 5–7 show the maximum ethanol titers, ethanol productivities, and residual carbohydrate levels for SSF and fermentation trials with hulls. Since the hulls contain primarily fiber, and lower levels of oligosaccharides than the other soybean fractions, we anticipated that ethanol production would be enhanced.  Figure 5 shows a statistically significant difference in ethanol production between SSF trials with versus without enzymes as well as increased ethanol production as the SLR increased (except between 15% and 20% SLR). *S. cerevisiae* outperformed *S. stipitis* at the higher SLRs, perhaps due to increased ethanol tolerance. Maximum concentrations obtained were 23.177 g/L ± 10.148 for *S. cerevisiae* and 14.501 g/L ± 6.748 for *S. stipitis*. As with the beans, the hulls were highly viscous at the higher SLRs, making proper mixing of the slurry very difficult, adding to the variability of the trials. 

Ethanol productivities were calculated at the time of maximum ethanol titer and the results (Figure 6) mirrored the trends noted for ethanol titers (higher productivities with enzymes present, at higher SLR's, and with *S. cerevisiae*). Ethanol productivities increased with the SLR (maxima of 0.322 g/L/h ± 0.022 for *S. cerevisiae* and 0.151 g/L/h ± 0.067 for *S. stipitis*, both with enzymes), though the difference between the 15% and 20% SLR was not significant. As expected, residual carbohydrate levels were higher in trials with enzymes, compared to nonenzyme trials (Figure 7). *S. stipitis* trials also accumulated higher levels of residual carbohydrates, which matches the reduced ethanol titers observed in Figure 5. Accumulation of stachyose and raffinose was also correlated with higher SLR's. 

**Figure 8:**
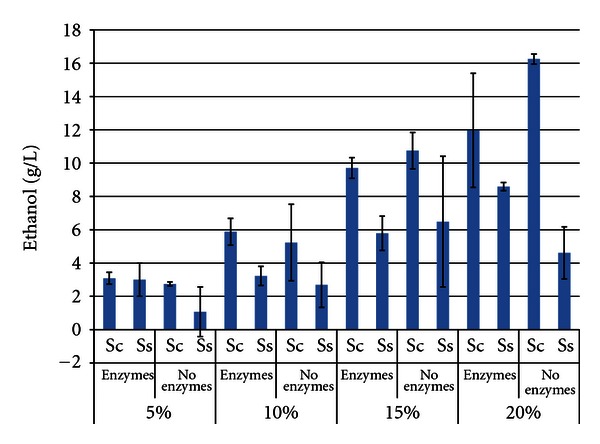
Maximum ethanol titer from white flakes after 96 h SSF or fermentation^1^. ^1^Error bars represent one standard deviation.

**Figure 9:**
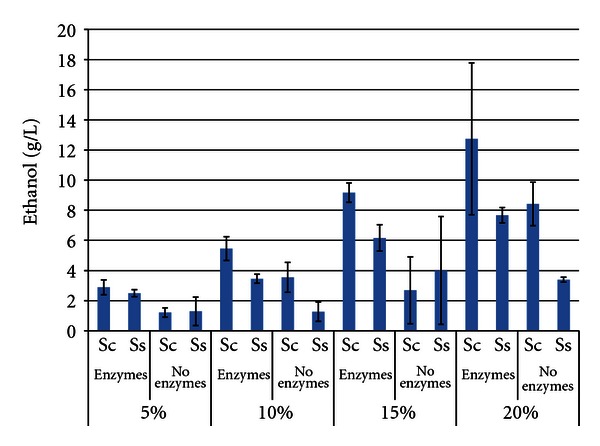
Maximum ethanol titer from soybean meal after 96 h SSF or fermentation^1^. ^1^Error bars represent one standard deviation.

**Figure 10:**
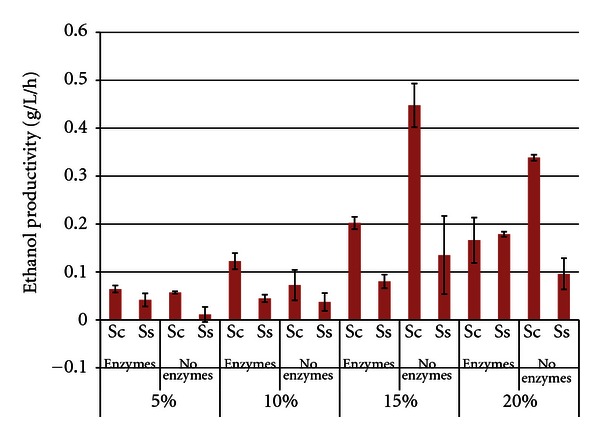
Ethanol productivity from white flakes after 96 h SSF or fermentation^1^. ^1^Error bars represent one standard deviation.

**Figure 11:**
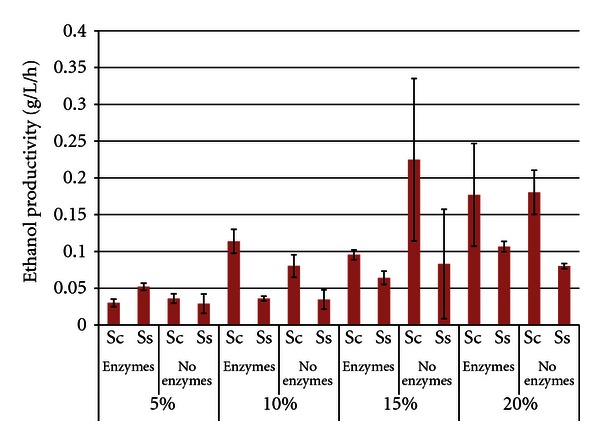
Ethanol productivity from soybean meal after 96 h SSF or fermentation^1^. ^1^Error bars represent one standard deviation.

**Figure 12:**
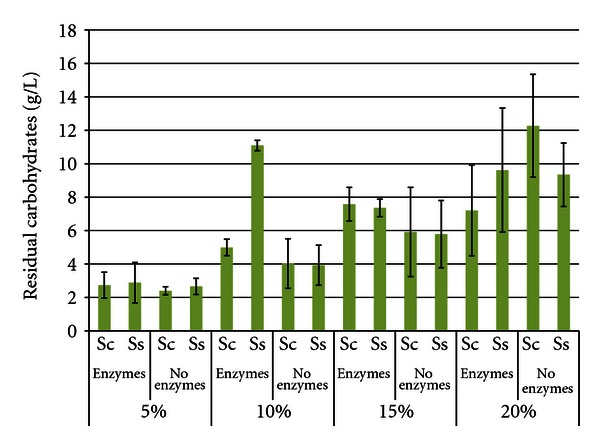
Residual carbohydrates from white flakes after 96 h SSF or fermentation^1^. ^1^Error bars represent one standard deviation.

**Figure 13:**
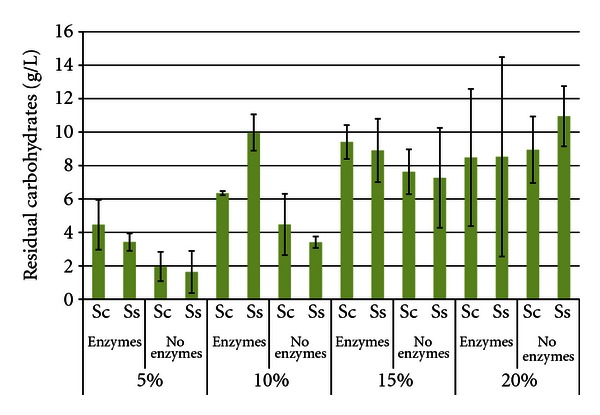
Residual carbohydrates from soybean meal after 96 h SSF or fermentation^1^. ^1^Error bars represent one standard deviation.

Figures 8–13 show the maximum ethanol titers, ethanol productivities, and residual carbohydrates levels for SSF and fermentation trials with white flakes and soybean meal. These fractions did not contain significant amounts of hulls (i.e., fiber); therefore, the primary carbohydrate source was oligosaccharides (stachyose and raffinose). The total carbohydrate levels in these fractions were much lower than in hulls, but higher than that in whole soybeans (due to the removal of oil). Thus, ethanol production was anticipated to be between that of soybeans and hulls. 

Figures 8 and 9 show the ethanol titers for white flakes and SBM, respectively. For both substrates, ethanol titers increased as the SLR increased. Addition of enzymes improved ethanol yields, but not to as great an extent as with hulls. This result was not surprising. *S. cerevisiae* once again produced more ethanol than *S. stipitis* with both substrates. For example, maximal ethanol titers for *S. cerevisiae* were 16.25 ± 0.30 g/L and 12.75 ± 5.03 g/L at 20% SLR without enzymes (white flakes) and with enzymes (SBM), respectively. *S. stipitis* had a corresponding maxima of 8.59 g/L ± 0.24 and 7.67 g/L ± 0.51 at 20% SLR with enzymes for white flakes and SBM, respectivly. Viscosity and mixing became an issue with both substrates at the higher SLR's, again affecting variability between replications. 

Figures 10 and 11 show the ethanol productivity from the white flake and SBM, SSF, and fermentation trials, respectively. As expected, ethanol productivity data trended similarly to ethanol titer data, with values increasing up to 15% SLR and then leveling off. This was likely due to the increased viscosity of the slurries at the higher SLR's. In trials with white flakes, maximum productivities were 0.45 g/L/h ± 0.05 for *S. cerevisiae* at 15% SLR without enzymes, and 0.18 g/L/h ± 0.01 for *S. stipitis* at 20% with enzymes for white flakes. In trials with SBM, maximum productivities were 0.23 g/L/h ± 0.11 for *S. cerevisiae* at 15% SLR without enzymes and 0.11 g/L/h ± 0.01 for *S. stipitis* at 20% SLR with enzymes. *S. cerevisiae* generally had higher ethanol productivities than *S. stipitis*, but high variability at the higher SLR's often prevented significant differences from being established.

Figures 12 and 13 show the residual carbohydrate levels from the white flakes and SBM, respectively. These results are highly variable with very few statistically significant differences between pairs of treatments. Residual carbohydrate levels increased as the SLR increased due to accumulation of unfermented oligosaccharides. In general, the presence of enzymes also resulted in the accumulation of more carbohydrates than in trials lacking enzymes. There was not as much difference between the yeasts in trials with soybeans and white flakes, but *S. cerevisiae* typically had lower residual sugar levels than *S. stipitis*. 

Table 5 shows the difference in residual carbohydrates in 10% SLR SSF or fermentation trials compared to the saccharification trials. The yeasts reduced the amount of total carbohydrates in all of the 10% SLR trials when compared to the amount of total carbohydrates in solution after the 96 h saccharification or enzyme-free comparisons. In the trials with enzymes, *S. cerevisiae* reduced the carbohydrate levels more than *S. stipitis*. In the trials without enzymes, the *S. stipitis* reduced the carbohydrates more than the *S. cerevisiae*, but only by very small amounts. These data suggest that *S. stipitis* was more susceptible to high osmotic pressure than *S. cerevisiae*, but was otherwise comparable to *S. cerevisiae* at consuming carbohydrates at a 10% SLR of any of these substrates. However, the ethanol titers for these trials also showed that *S. cerevisiae* was capable of producing more ethanol than *S. stipitis.*

Table 6 shows the fiber, protein, and lipid differences of the four substrates after 96 h SSF, compared to their initial values. These data came from the 10% SLR trials and replicate samples were combined before analysis. All trials, except for SBM without enzymes, showed a decrease in fiber levels, with enzyme-treated substrates showing an average decrease of 79.9% compared to trials without enzymes (avg fiber decrease of 11.4%). 

We had anticipated that reducing fiber levels (and converting some of the resultant sugars into biomass) would increase protein levels. In the case of enzyme-treated hulls, we observed an average 54.2% increase in protein content. Mielenz et al. [29] showed an even greater rise in protein concentration, 2.5 times that of the starting material. Mielenz et al. optimized the enzyme dosages and conducted SSF for 13 days, which if adopted in our study would have resulted in greater conversion of fibers into fermentable sugars and subsequently ethanol. However, fermentation times in excess of 4 days are typically not economically practical. In our other treatments, the protein level actually decreased by an average of 10.7% (range 2.46%–20.2%). This loss could be explained by protein catabolism or by the large gain in lipid levels reducing the concentrations of other components (such as protein). 

Lipid concentrations increased by an average of 37% in soybean trials, likely due to the conversion of fibers into sugars and then ethanol. The remaining samples all showed a large percentage increase in lipid (average ~764%), but these numbers are somewhat misleading unless one considers the low content of lipids in hulls, white flakes, and SMB (avg ≤ 1.5% oil) in these substrates. Lipids from yeast cell mass production likely accounted for this change.

## 4. Conclusions

Ethanol production as a means of removing carbohydrates from soybean fractions to concentrate the remaining components was successful in some cases. For example, SSF treatment of soybeans reduced fiber levels by ~84% and concentrated oil by ~46% in the fermented solids. Similar treatment of soybean hulls reduced fiber levels by ~94% and concentrated protein by ~54%. Fiber levels were also significantly lowered in white flake and SBM fractions and oil levels were raised (although the percentage increases are somewhat misleading due to the low levels of oil present initially in these fractions). These changes would have been more impressive if the yeasts were capable of utilizing the xylose present. 

As noted previously, Mielenz et al. [29] treated a 20% solid loading rate slurry of soybean hulls with a cocktail of cellulase, *β*-glucosidase, and pectinase in a 13-day SSF process. They achieved ethanol titers of 32.5 g/L and increased the protein content by 2.5–3.0 times the original concentration of the substrate. However, Schirmer-Michel et al. [30] were only able to produce ~5.7 g/L ethanol and on 15 g/L xylose loading rate slurry of acid-hydrolyzed soybean hulls using *Candida guilliermondii* NRRL Y-2075. 

Future research should seek to minimize enzyme dosage requirements, while using yeast strains able to convert all of the monosaccharides into ethanol. Minimizing soy protein catabolism by yeast is another critical parameter and this could be achieved by minimizing SSF time. 

## Figures and Tables

**Table 1:** Proximate analysis of soybean substrates^1^.

	Soybeans	Soybean hulls	Soybean white flakes	Soybean meal
Crude protein, % (AOAC 992.23)	38.71	11.71	51.16	52.60
Crude fat (diethyl ether extraction), %	N/A	1.56	0.939	0.797
Crude fat (double extraction), %	20.20	N/A	N/A	N/A
Ash-total, % (AOAC 942.05)	5.15	5.09	6.43	6.54
Crude fiber, % (AOAC 948.10)	9.01	40.30	4.99	3.86
Nitrogen free extract, %	26.90	41.40	36.50	36.20

^1^Dry matter basis.

**Table 2:** Glucan content of soybean substrates.

Substrate	[18]	[21]	[22]	[1]	[23]	[24]	Avg%
Soybeans		5.8	5.1				5.27
			4.9				
Soybean meal				3.0	3.7		4.28
				5.0	5.4		
Soybean hulls		40.1		46.0		45.0	43.7
Soybean white flakes	3.1						3.1

**Table 3:** Enzyme volumes for saccharification and 5% SSF trials.

Substrate	Solids (g)	Fiber (g)	NS50013 (mL)	NS50010 (mL)	NS22016 (mL)
Soybeans	15	0.79	0.22	0.11	0.1
Soybean meal	15	0.64	0.18	0.09	0.08
Soybean hulls	15	6.56	1.84	0.89	0.87
Soybean white flakes	15	0.47	0.13	0.06	0.06

**Table 4:** Carbohydrates, fiber, protein, and lipid concentrations after 96 h saccharification.

	Beans		Meal	
	With enzymes	Without enzymes	With enzymes	Without enzymes
Soluble carbohydrates (g/L)	14.90 ± 2.04	10.45 ± 1.61	47.24 ± 5.83	35.08 ± 1.96
Fiber difference (%)^1^	−45.60	−25.86	−27.72	3.37
Protein difference (%)^1^	−12.12	−12.30	−10.30	−8.73
Lipid difference (%)^1^	12.87	4.46	−17.94	−31.99

	Hulls		White flakes	
	With enzymes	Without enzymes	With enzymes	Without enzymes

Soluble carbohydrates (g/L)	58.23 ± 4.03	16.88 ± 1.17	37.92 ± 6.64	31.32 ± 4.96
Fiber difference (%)^1^	−80.69	−8.93	−76.36	−2.40
Protein difference (%)^1^	11.27	−13.75	−11.00	10.63
Lipid difference (%)^1^	−44.23	−12.18	−33.87	72.52

^1^Compared to the original substrate on a dry matter basis.

**Table 5:** Residual carbohydrate differences from saccharification trials to 10% SSF trials.

Substrate	Enzymes		No enzymes	
	*S. cerevisiae*	*S. stipitis*	*S. cerevisiae*	*S. stipitis*
Beans	−6.65	−2.76	−3.81	−4.41
Hulls	−37.00	−32.58	−9.91	−10.1
White flakes	−29.93	−23.82	−24.29	−24.39
Meal	−37.89	−34.27	−27.61	−28.67

**Table 6:** Fiber, protein, and lipid difference of substrates at 10% solid loading after 96 h SSF or fermentation.

			Fiber difference (%)^1^	Protein difference (%)^1^	Lipid difference (%)^1^
Beans	Enzymes	Sc	−80.0	−8.42	49.1
		Ss	−87.4	−8.6	42.8
	No enzymes	Sc	−23.4	−8.91	30.2
		Ss	−12.5	−8.65	25.7

Hulls	Enzymes	Sc	−94.19	55.0	667.31
		Ss	−93.92	53.37	717.31
	No enzymes	Sc	−10.92	−20.2	527.56
		Ss	−10.67	−18.49	506.41

WF	Enzymes	Sc	−78.76	−2.46	682.75
		Ss	−48.3	−10.4	984.13
	No enzymes	Sc	−20.26	−16.83	1038.45
		Ss	−13.83	−14.56	940.47

Meal	Enzymes	Sc	−72.3	−8.14	1224.48
		Ss	−84.5	−6.06	1283.94
	No enzymes	Sc	−1.04	−10.8	593.85
		Ss	1.3	−7.55	548.68

^1^Compared to original substrate on dry matter basis.
